# Association between Frequency of Consumption of Fruit, Vegetables, Nuts and Pulses and BMI: Analyses of the International Study of Asthma and Allergies in Childhood (ISAAC)

**DOI:** 10.3390/nu10030316

**Published:** 2018-03-07

**Authors:** Clare R. Wall, Alistair W. Stewart, Robert J. Hancox, Rinki Murphy, Irene Braithwaite, Richard Beasley, Edwin A. Mitchell, The ISAAC Phase Three Study Group

**Affiliations:** 1 Discipline of Nutrition and Dietetics, Faculty of Medicine and Health Sciences, University of Auckland, Auckland 1010, New Zealand; 2 School of Population Health, University of Auckland, Auckland 1010, New Zealand; a.stewart@auckland.ac.nz; 3 Department of Preventive & Social Medicine, Dunedin School of Medicine, University of Otago, Dunedin 9054, New Zealand; bob.hancox@otago.ac.nz; 4 Department of Medicine, Faculty of Medicine and Health Sciences, University of Auckland, Auckland 1010, New Zealand; r.murphy@auckland.ac.nz; 5 Medical Research Institute of New Zealand, Wellington 6021, New Zealand; Irene.Braithwaite@mrinz.ac.nz (I.B.); Richard.Beasley@mrinz.ac.nz (R.B.); 6 Department of Paediatrics: Child and Youth Health, Faculty of Medicine and Health Sciences, The University of Auckland, Auckland 1010, New Zealand; e.mitchell@auckland.ac.nz

**Keywords:** BMI, children, adolescents, nuts, fruit, vegetables, pulses, ISAAC

## Abstract

Diets which emphasize intakes of plant-based foods are recommended to reduce disease risk and for promoting healthy weight. The aim of this study was to examine the association between fruit, vegetables, pulses and nut intake and body mass index (BMI) across countries in adolescents (13–14 years) and children (6–7 years). Data from the International Study of Asthma and Allergies in Childhood; 77,243 children’s parents and 201,871 adolescents was used to examine the association between dietary intake (Food Frequency Questionnaire) and BMI using general linear models, adjusting for country gross national index. Adolescents who consumed fruit, vegetables, pulses and nuts three or more times a week had a lower BMI than the never or occasional group; eating nuts three or more times a week, was associated with a BMI value of 0.274 kg/m^2^ lower than the never group (*p* < 0.001). Compared to children who never or occasionally reported eating vegetables, those reporting that they ate vegetables three or more times per week had a lower BMI of −0.079 kg/m^2^. In this large global study, an inverse association was observed between BMI and the reported increasing intake of vegetables in 6–7 years old and fruit, vegetables, pulses and nuts in adolescents. This study supports current dietary recommendations which emphasize the consumption of vegetables, nut and pulses, although the effect sizes were small.

## 1. Introduction

Approximately 41 million children younger than five years are overweight or obese [1]. Global prevalence data for older children is not available but studies from Europe show increases in obesity rates over the last two decades [2]. The WHO Report of the Commission on Ending Childhood Obesity (WHO) has recognised the severity of this global epidemic and its recommendations reflect the fact that childhood obesity is a result of an energy imbalance which has resulted from changes in food type, availability, affordability, and marketing, as well as a decline in physical activity, with more time being spent on sedentary leisure activities [1].

In the quest to reduce the burden of chronic disease and obesity many countries and authorities have adopted an evidence-based approach to population dietary guidelines [3,4]. This emphasises the importance of a plant-based diet and encourages increasing intake of fruits and vegetables and foods which increases dietary fibre intake such as whole grains, legumes, pulses and nuts. A diet rich in fruits, vegetables and wholegrain foods may help in reducing the risk of obesity because fruits and vegetables have high content of water and dietary fibre which may increase satiety [5]. The demonstration of consistent trends in the associations with BMI in childhood and protective dietary patterns across countries with different cultures would provide further evidence to support these guidelines.

The International Study of Asthma and Allergies in Childhood (ISAAC) Phase Three is a multi-national multi-centre study that has previously collected data on heights and weights of children aged 13–14 years (adolescents) and 6–7 years as well as dietary intake [6]. The study was originally designed to measure time trends in the prevalence and severity of asthma, rhino -conjunctivitis and eczema and to explore the relationship between lifestyle, other putative risk factors and the development of asthma and allergies. However, ISAAC Phase Three has also provided us with the opportunity to examine the association between fruit, vegetables, pulses and nut intake and body mass index (BMI) across 36 countries in adolescents and 18 countries in children.

## 2. Methods

ISAAC centres were from defined geographical areas (centres) within countries. A minimum of 10 schools randomly sampled (or all schools used) within each centre for each age group. Two age groups were chosen—the compulsory age group was the adolescent group who self-completed the questionnaire at school and highly recommended were the children, whose parents/guardians completed the questionnaire about their child at home. ISAAC Phase Three used the Phase One standardised core questionnaire on symptoms of asthma, rhino conjunctivitis and eczema, and included a recommended environmental questionnaire (EQ) to collect potential risk factor data which included questions on participant dietary intake, as well as height and weight. Phase Three was undertaken between 2001 and 2003. The questionnaires are on the ISAAC website and can be accessed at the following url: http://isaac.auckland.ac.nz. As this is a secondary analysis of pre-existing non-identifiable data, ethics approval was not sought for this study, although approval from the principal investigator in each centre was obtained.

### 2.1. Main Outcome Variable—Body Mass Index

Height and weight were reported by the adolescents and by the parents of the children. In some centres, each subject’s height and weight were measured objectively although there were no standardised instructions for doing this. BMI was calculated as weight (kg)/(height (m))^2^. For 20% of children and 23% of adolescents the height and weight was measured objectively. To eliminate likely erroneous data, we applied the following rules:

For adolescents in each centre, those in the top and bottom 0.5% of weights and heights, and those with heights less than 1.25 m were excluded. BMIs less than 10 kg/m^2^ and greater than 45 kg/m^2^ were removed. For children in each centre, those in the top and bottom 0.5% of weights and heights, and those with heights less than 1.0 m were excluded. BMIs less than 9 kg/m^2^ and greater than 40 kg/m^2^ were removed.

### 2.2. Explanatory Variables

Questions about dietary intake of foods were asked using a questionnaire which was developed from an ecological analysis of the relationship between per capita food intake and the prevalence of symptoms of asthma, allergic rhino conjunctivitis and atopic eczema [7]. The questions on foods were suitable for all ethnic groups and the questions asked ‘In the past 12 months, how often, on average, did you (did your child) eat or drink the following: meat; seafood; whole fruit (not juices); vegetables (green and root); pulses (peas, beans, lentils); cereal; pasta (including bread); rice; butter; margarine; nuts; potatoes; milk; eggs and fast food/burgers?’. Centres were encouraged to include local names to define foods if necessary. The three categories for food intake were: never or occasionally; once or twice per week; and ≥3 times per week. Questionnaires were translated into 53 languages, back-translated into English and assessed for accuracy [8]. For the purpose of this paper foods were used that we hypothesized would be associated with a lower BMI. The data on the intake of fruit, vegetables (green and root), pulses (peas, beans, lentils) and nuts were examined. We have previously reported on the association of fast food and BMI in this study [9].

We also adjusted for gross national income (GNI) by country as this has been shown to be associated with childhood obesity. Country gross national income (GNI) was based on the 2006 World Bank categories of high-, high middle-, low middle- and low-income countries, dichotomised into high income (high- plus high middle-income) and low-income (low middle- plus low-income) categories.

### 2.3. Statistical Analysis

BMI was assessed separately for each age group using a general linear mixed model (GLMM) with centre as a random effect and GNI for each country, the individual’s age, sex, measurement type, and dietary intake variable as fixed effects. SAS (v9.3, SAS Institute, Cary, NC, USA) was used. The regression coefficients from the GLMM for use once or twice per week and use three or more times per week relative to no use or occasional use are reported in the table along with the P value for the combined effect of these categories.

## 3. Results

**Figure 1:**
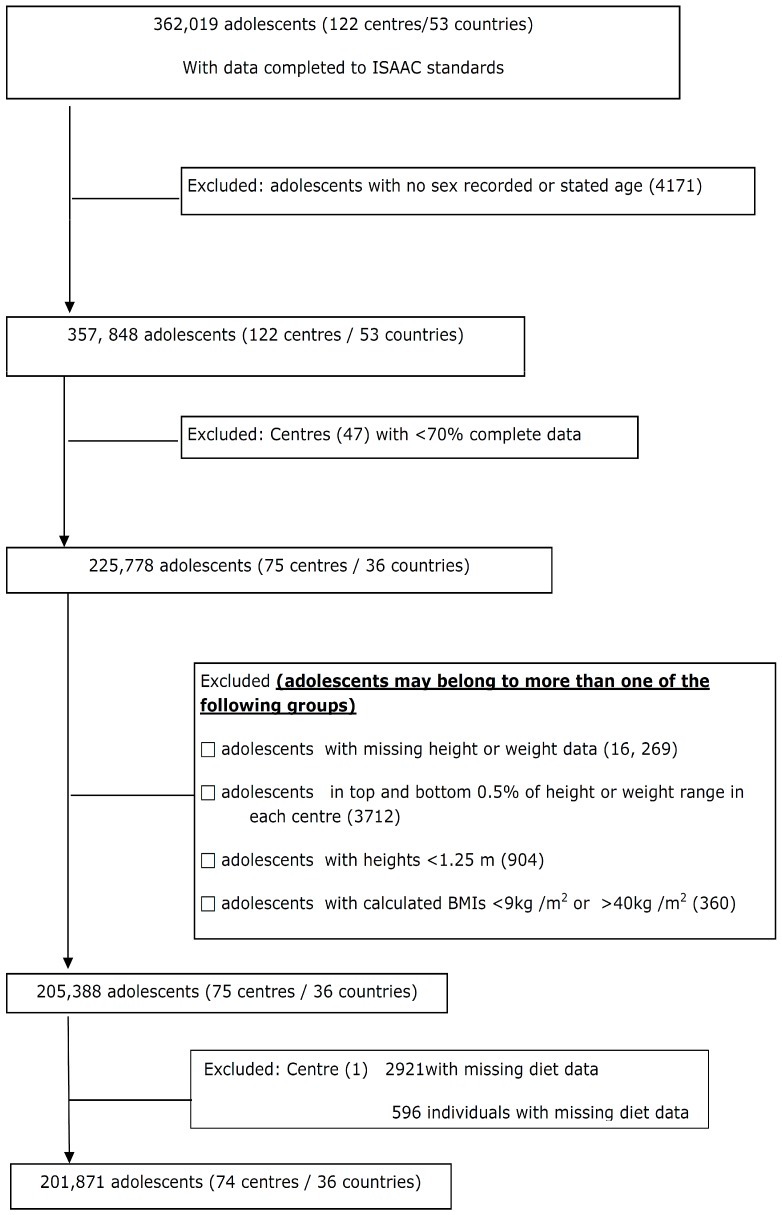
Flow of adolescents through the study.

For the adolescents, data were submitted from 122 centres in 53 countries (362,019 subjects). Centres that submitted data which included heights, weights, diet information, and met the inclusion criteria comprised 201,871 adolescents (74 centres/36 countries) who were included in the final analysis (Figure 1).

**Figure 2:**
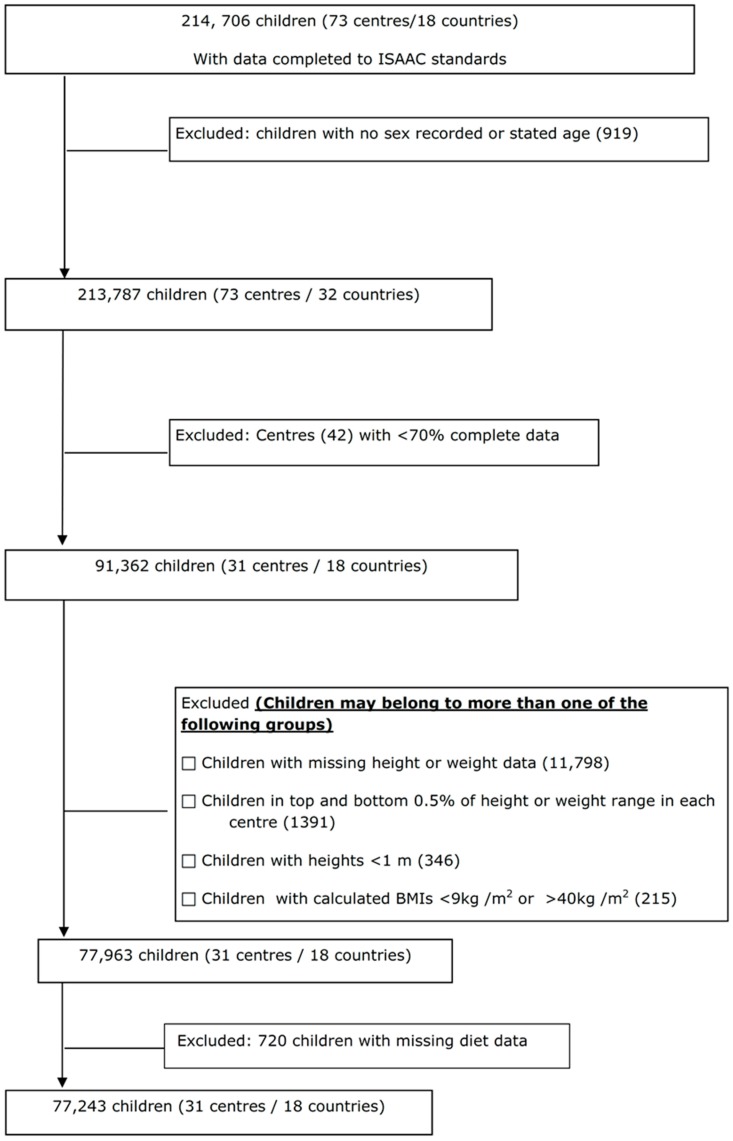
Flow of children through the study.

For the children, data were submitted from 73 centres in 32 countries (214,706 subjects). Centres that submitted data which included heights, weights, diet information, and met the inclusion criteria comprised 77,243 children (31 centres/18 countries) who were included in the final analysis (Figure 2).

After controlling for country GNI, centre, age, sex, measurement type (measured and reported), The adolescents in the three or more times and the 1–2 times a week group for fruit, vegetables, pulses and nuts had a significantly lower BMI than the never group. The strongest association of intake with BMI was observed in adolescents who ate nuts three or more times a week, which was associated with a BMI values −0.274 (0.024) kg/m^2^ lower than the never group, whereas the 1–2 times a week group had BMI values −0.176 (0.016) kg/m^2^ lower than the never group (*p* <0.001). The children who consumed vegetables three or more times a week had a lower BMI −0.079 (0.031) kg/m^2^ than the never group whereas the 1–2 times a week group had BMI −0.057 (0.031) kg/m^2^ lower than the never group (Table 1). Both of these were statistically significant (*p* = 0.03).

**Figure 3:**
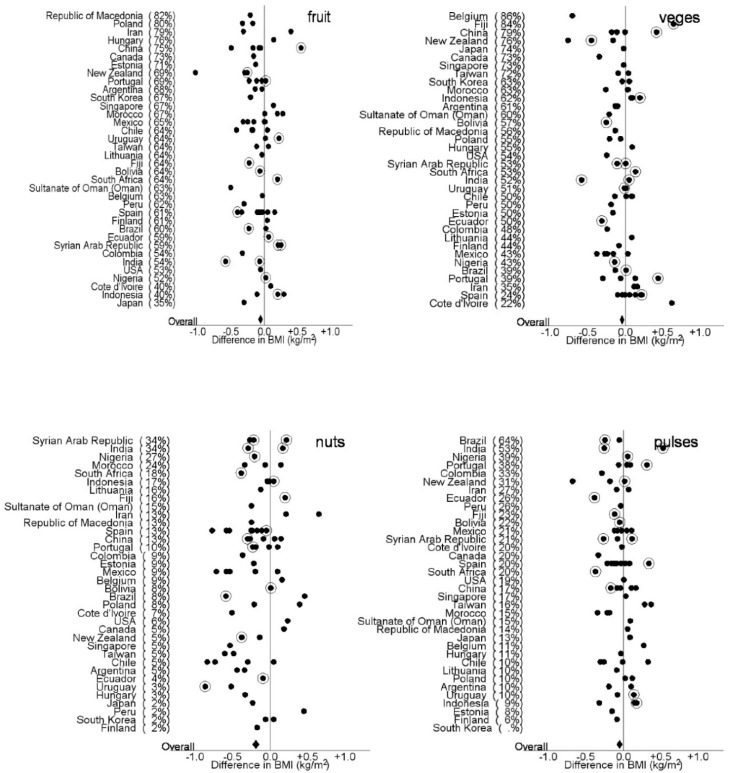
Differences in BMI (kg/m^2^) between adolescents who reported eating fruit, vegetables, pulses, and nuts three or more times a week and those children who reported eating fruit, vegetables, pulses and nuts 1–2 times per week or never, in each centre after controlling for country GNI, centre, age, sex, measurement type (reported or measured). The solid diamond represents the overall effect. The proportion of participants who reported eating fruit, vegetable, pulses and nuts three or more times per week is shown in parentheses after each country. The solid dots represent centres with reported heights and weights and the circled dots represent centres that measured heights and weights.

**Figure 4:**
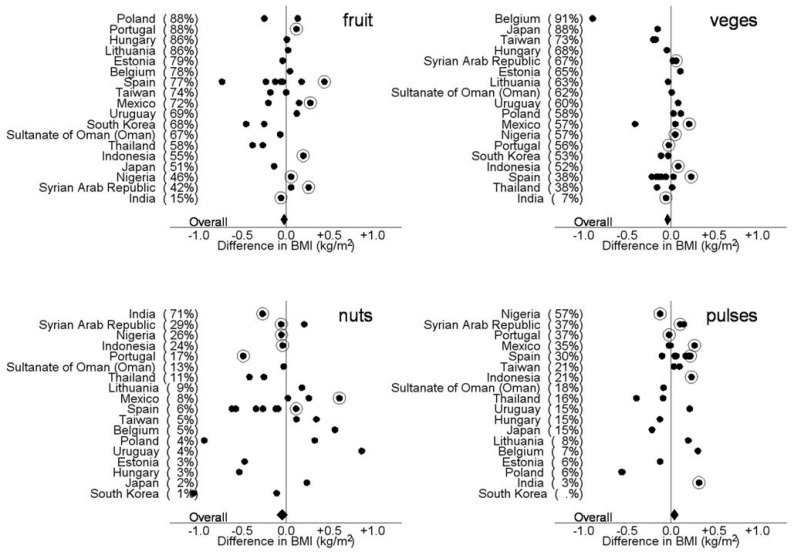
Differences in BMI (kg/m^2^) between children who reported eating fruit, vegetables, pulses, and nuts three or more times per week and those who reported eating fruit, vegetables, pulses and nuts 1 to 2 times per week or never in each centre after controlling for country GNI, age, sex, measurement type (reported or measured). The solid diamond represents the overall effect. The proportion of participants who reported eating fruit, vegetable, pulses and nuts three or more times per week is shown in parentheses after each country. The solid dots represent centres with reported heights and weights and the circled dots represent centres that measured heights and weights.

Figure 3 and Figure 4 show the differences in BMI (kg/m^2^) between adolescents and children who reported eating fruit, vegetables, pulses, and nuts three or more times per week and those who reported eating fruit, vegetables, pulses, 1–2 times per week or never in each centre after controlling for country GNI, age, sex, measurement type (reported or measured). The proportion of participants who reported eating fruit, vegetable, pulses and nuts three or more times per week is shown in parentheses after each country. The figures demonstrate the diversity of intake of fruit, vegetables, pulses and nuts between countries and centres and the direction of the effect of greater intake on BMI. In the adolescents there was an overall negative association with eating three or more fruit, vegetables, pulses and nuts per week on BMI. In the children there was an overall negative association with eating three or more fruit, vegetables and nuts per week on BMI and a positive association with of eating three or more pulses per week.

## 4. Discussion

This study of 201,871 adolescents in 74 centres in 36 countries and 77,243 children in 31 centres in 18 countries and demonstrates an inverse association between BMI and the consumption of vegetables in children and fruit, vegetables, pulses and nuts in adolescents.

There have been few studies which have examined dietary intake across many countries and BMI in children. A previous study of 34 (primarily European) participating countries of the 2001–2002 Health Behaviour in School-Aged Children Study reported that overweight status was not associated with the intake of fruits or vegetables [10]. A US prospective cohort study of 14,900 children demonstrated that neither fruit nor fruit juice intake predicted changes in BMI, but among the boys, vegetable intake was inversely related to changes in BMI z-score [11]. In our study we saw a small inverse association between BMI and eating fruit and vegetables, three or more times a week but were unable to examine total dietary energy intake in relation to fruit and vegetable intake. However, a systematic review and meta-analyses of eight studies which have examined the effect of promoting increased fruit and vegetable consumption in adults, in the absence of specific advice to decrease consumption of other foods (and energy intake), suggests that this may be useful for weight maintenance or loss [12].

Studies of children who adhere to vegetarian diets or dietary patterns which are rich in vegetables, whole grains, nuts, pulses, and legumes have shown consistent protective effects of these diets on the risk of being overweight [13]. Similarly, cohort studies in adults have shown that dietary patterns which are more closely aligned to the Mediterranean diet (rich in vegetables, legumes, fruit and nuts, cereals, fish and seafood, olive oil, and moderate amounts of alcohol) help to protect against weight gain and subsequent obesity [14].

Our study established that there is an inverse association between eating nuts and BMI in both adolescents and children with the estimates being significantly larger for the adolescent group who consumed nuts three or more times a week. This finding of the inverse association of consumption of nuts on BMI is consistent with previous clinical trials and epidemiological studies of the effects of nut consumption in adults [15,16]. There are several plausible reasons which may explain why nut consumption is associated with a lower BMI. These include: enhanced satiety, incomplete absorption of fat from nuts, increased resting metabolic rate, and corresponding decreased intake of other foods. Nuts are energy dense due to their high fat content; they are a good source of protein and are low in saturated fat [16]. Nuts are also high in dietary fibre which increases satiety and suppresses appetite [17]. Incomplete absorption of fat from nuts due to incomplete mastication also causes a loss of available energy due to faecal fat loss [18,19].

Although an inverse relationship between BMI and vegetables in children and fruit, vegetables, pulses and nuts in adolescents was observed there were fewer adolescents consuming fruit and vegetables more than three times a week. This is not surprising and could reflect factors affecting food intake between the two age groups, where adolescents may have more independence, money and more control over their diet than younger children [20]. It could also reflect the difference in reporting as the adolescent data was self-reported and the children’s dietary intake data was obtained through parental report.

Although this cross-sectional study constitutes a large global data set, the collection of dietary intake data in this study was kept as brief as possible with a limited number of food items and only three categories for frequency of food intake to enable greater global participation. The data are therefore only useful for ranking participants for specific food items so that characteristics of those with high and low intakes can be compared. This has been completed in previous analyses of the ISAAC data where the association between fast foods and BMI and the symptom prevalence of asthma, rhino conjunctivitis and eczema have been explored [9,21]. However it must be noted that quantification of total intake based on frequency alone will be inaccurate because of the absence of information on portion sizes. Another limitation of this study is that weights and heights were self-reported in most centres by the parents or adolescents. Although this raises questions about the accuracy of the BMI values, which may be further compounded by selection bias across the various countries examined, others have shown that self-reported heights and weights are fairly reliable [22]. Also in our analyses we did not use a standardised BMI scale (z score) or BMI cut-off points to define overweight or obesity because we had a very small age range (either 6–7 or 13–14) and using standardisation might cover or exaggerate differences of interest. It should also be noted that this is a secondary analysis of existing data and with limited information available to us few variables are included in our model. It is therefore possible that our findings are as a result of confounding with other factors. 

Our results suggest that there is an inverse association of BMI with a greater consumption of, fruit, vegetables, pulses and nuts in adolescents and vegetables in 6–7-year-old. Although causality cannot be proven in an observational study the findings support current global dietary recommendations that emphasize regular consumption of fruit, vegetables (green and root) and nuts for the prevention of overweight and obesity.

## Figures and Tables

**Table 1:** BMI ^i^ difference relative to the never use category for reported dietary intake of fruit, vegetables, pulses and nuts and BMI of study participants (+/− kg/m^2^, (SE)).

		Use Once or Twice per Week	Use Three or More Times per Week	*p* Value
Adolescents (*N* = 199,723)	Fruit	−0.061 (0.028)	−0.104 (0.026)	<0.001
Adolescents (*N* = 198,692)	Vegetables	−0.014 (0.023)	−0.053 (0.023)	0.01
Adolescents (*N* = 188,734)	Pulses	−0.104 (0.017)	−0.119 (0.022)	<0.001
Adolescents (*N* = 196,948)	Nuts	−0.176 (0.016)	−0.274 (0.024)	<0.001
Children (*N* = 76,657)	Fruit	−0.061 (0.036)	−0.051 (0.038)	0.24
Children (*N* = 75,684)	Vegetables	−0.057 (0.031)	−0.079 (0.031)	0.03
Children (*N* = 70,473)	Pulses	0.002 (0.024)	0.042 (0.029)	0.21
Children (*N* = 74,574)	Nuts	−0.029 (0.020)	−0.056 (0.033)	0.14

^i^ Adjusted regression coefficients—adjusted for age, sex, measurement type and centre.

## ISAAC Steering Committee

N Aït-Khaled * (International Union Against Tuberculosis and Lung Diseases, Paris, France); HR Anderson (Division of Community Health Sciences, St Georges, University of London, London, UK); MI Asher (Department of Paediatrics: Child and Youth Health, Faculty of Medical and Health Sciences, The University of Auckland, New Zealand); R Beasley * (Medical Research Institute of New Zealand, Wellington, New Zealand); B Björkstén * (Institute of Environmental Medicine, Karolinska Institutet, Stockholm, Sweden); B Brunekreef (Institute of Risk Assessment Science, Universiteit Utrecht, Netherlands); J Crane (Wellington Asthma Research Group, Wellington School of Medicine, New Zealand); P Ellwood (Department of Paediatrics: Child and Youth Health, Faculty of Medical and Health Sciences, The University of Auckland, New Zealand); C Flohr (Department of Paediatric Allergy and Dermatology, St Johns Institute of dermatology, St Thomas’ Hospital, London, UK); S Foliaki * (Centre for Public Health Research, Massey University, Wellington, New Zealand); F Forastiere (Department of Epidemiology, Local Health authority Rome, Italy); L García-Marcos (Respiratory Medicine and Allergy Units, ‘Virgen de la Arrixaca’ University Children’s Hospital, University of Murcia, Spain); U Keil * (Institut für Epidemiologie und Sozialmedizin, Universität Münster, Germany); CKW Lai * (Department of Medicine and Therapeutics, The Chinese University of Hong Kong, SAR China); J Mallol * (Department of Paediatric Respiratory Medicine, University of Santiago de Chile, Chile); EA Mitchell (Department of Paediatrics: Child and Youth Health, Faculty of Medical and Health Sciences, The University of Auckland, New Zealand); S Montefort * (Department of Medicine, University of Malta, Malta), J Odhiambo (deceased) (Centre Respiratory Diseases Research Unit, Kenya Medical Research Institute, Nairobi, Kenya); N Pearce (Department of Medical Statistics, Faculty Epidemiology and Public Health, London School of Hygiene and Tropical Medicine, London, UK); CF Robertson (Murdoch Children’s Research Institute, Melbourne, Australia); AW Stewart (Population Health, Faculty of Medical and Health Sciences, The University of Auckland, New Zealand); D Strachan (Division of Community Health Sciences, St Georges, University of London, London, UK); E von Mutius (Dr von Haunerschen Kinderklinik de Universität München, Germany); SK Weiland (deceased) (Institute of Epidemiology, University of Ulm, Germany); G Weinmayr (Institute of Epidemiology and Medical Biometry, University of Ulm, Germany); H Williams (Centre for Evidence Based Dermatology, Queen’s Medical Centre, University Hospital, Nottingham, UK); G Wong (Department of Paediatrics, Prince of Wales Hospital, Hong Kong, SAR China).

## ISAAC International Data Centre

MI Asher, TO Clayton (deceased), E Ellwood, P Ellwood, EA Mitchell, Department of Paediatrics: Child and Youth Health, and AW Stewart, School of Population Health, Faculty of Medical and Health Sciences, The University of Auckland, New Zealand.

## ISAAC Phase Three Study Group

ISAAC Principal Investigators: **Argentina:** Dr M Gómez, Ayre Foundation; Hospital San Bernardo (Salta); **Belgium:** Professor J Weyler, University of Antwerp (Antwerp); **Bolivia:** Dra R Pinto-Vargas *, Caja Petrolera de Salud (Santa Cruz); **Brasil:** Professor AJLA Cunha, Federal Universtity of Rio de Janeiro (Nova Iguaçu), Associate Professor L de Freitas Souza, Universidade Federal da Bahia (Vitória da Conquista); **Canada:** Professor A Ferguson, University of British Columbia (Vancouver); **Chile:** Dr P Aguilar, Hospital CRS El Pino (South Santiago), Dr L Amarales, Regional Hospital “Lautaro Navarro” (Punta Arenas), Dr LAV Benavides, (Calama), Dra A Contreras, Hospital de Castro (Chiloe); **China:** Professor Y-Z Chen *, Training Hospital for Peking University (Beijing, Tong Zhou), Assistant Professor O Kunii, University of Tokyo (Tibet), Dr Q Li Pan, Xinjiang Children’s Hospital (Wulumuqi); Professor NS Zhong, Guangzhou Institute of Respiratory Disease (Guangzhou); **Colombia:** Dr AM Cepeda, Universidad Metropolitana (Barranquilla); **Cote d’Ivoire:** Dr BN Koffi *, (Urban Cote d Ivoire); **Ecuador:** Dr C Bustos, Hospital Alcivar (Guayaquil); **Estonia:** Dr M-A Riikjärv *, Tallinn Children’s Hospital (Tallinn); **Fiji:** Dr R Sa’aga-Banuve, UNICEF (Suva); **Finland:** Dr J Pekkanen *, National Public Health Institute (Kuopio County); **Former Yugoslav Republic of Macedonia (FYROM):** Associate Professor E Vlaski *, University Children’s Clinic (Skopje); **Hungary:** Dr G Zsigmond *, Senior Consultant (Svábhegy); **India:** Dr SN Mantri, Jaslok Hospital & Research Centre (Mumbai (29)), Professor SK Sharma, All India Institute of Medical Sciences (New Delhi (7)); **Indonesia:** Prof Dr CB Kartasasmita, Padjajaran University (Bandung), Professor P Konthen (deceased), Airlangga University (Bali), Dr W Suprihati, Diponegoro University (Semarang); **Iran:** Dr M-R Masjedi *, National Research Institute of Tuberculosis and Lung Diseases (Rasht, Tehran); **Japan:** Dr H Odajima, National Hospital Organization Fukuoka Hospital (Fukuoka); **Lithuania:** Associate Professor J Kudzyte *, Kaunas Medical University (Kaunas); **Mexico:** Dra M Barragán-Meijueiro, CoMAAIPE (Ciudad de México (3)), Dra BE Del-Río-Navarro, Hospital Infantil de México (Ciudad de México (1)), Dr FJ Linares-Zapién, Centro De Enfermedades Alergicas Y Asma de Toluca (Toluca), Dra N Ramírez-Chanona, COMPEDIA (Ciudad de México (4)), Dr S Romero-Tapia, Hospital de Alta Especialidad del Niño (Villahermosa); **Morocco:** Professor Z Bouayad *, Service des Maladies Respiratoires (Boulmene, Casablanca, Marrakech); **New Zealand:** Dr R MacKay, Canterbury Health Laboratories (Nelson), Dr C Moyes, Whakatane Hospital (Bay of Plenty), Associate Professor P Pattemore, University of Otago, Christchurch (Christchurch); **Nigeria:** Professor BO Onadeko, (Ibadan); **Peru:** Dr P Chiarella *, Universidad Peruana de Ciencias Aplicadas, UPC (Lima); **Poland:** Associate Professor A Brêborowicz, University of Medical Sciences (Poznan), Associate Professor G Lis *, Jagiellonian University (Kraków); **Portugal:** Dra R Câmara, Centro Hospitilar do Funchal (Funchal), Dr JM Lopes dos Santos, Hospital Pedro Hispano (Porto), Dr C Nunes, Center of Allergy and Immunology of Algarve (Portimao), Dr J Rosado Pinto *, Hospital da Luz (Lisbon); **Singapore:** Associate Professor DY Goh, National University of Singapore (Singapore); **South Africa:** Professor HJ Zar *, University of Cape Town (Cape Town); **South Korea:** Professor H-B Lee *, Hanyang University College of Medicine (Provincial Korea, Seoul); **Spain:** Professor A Blanco-Quirós, Facultad de Medicina (Valladolid), Dr RM Busquets, Universidad Autonoma de Barcelona (Barcelona), Dr I Carvajal-Urueña, Centro de Salud de La Ería (Asturias), Dr G García-Hernández, Hospital Universitario 12 de Octubre (Madrid), Professor L García-Marcos *, University of Murcia and IMIB-Arrxaca Research Institute (Cartagena), Dr C González Díaz, Universidad del País Vasco UPV /EHU (Bilbao), Dr A López-Silvarrey Varela, Fundacion Maria Jose Jove (A Coruña), Professor M Morales-Suárez-Varela, Valencia University-CIBERESP (Valencia), Professor EG Pérez-Yarza, Universidad del Pais Vasco UPV/EHU (San Sebastián); **Sultanate Of Oman:** Professor O Al-Rawas *, Sultan Qaboos University (Al-Khodh); **Syrian Arab Republic:** Dr S Mohammad *, Tishreen University (Tartous), Professor Y Mohammad, National Center for Research and Training in Chronic Respiratory Diseases—Tishreen University (Lattakia), Dr K Tabbah, Aleppo University Hospital (Aleppo); **Taiwan:** Dr J-L Huang *, Chang Gung University (Taipei), Dr C-C Kao, Kao-Chun-Chieh Clinic (Taoyuan); **Thailand:** Associate Professor M Trakultivakorn, Chiang Mai University (Chiang Mai), Dr P Vichyanond *, Mahidol University (Bangkok); **Usa:** Dr HH Windom, Asthma and Allergy Research Center (Sarasota); **Uruguay:** Dra D Holgado *, Hospital Pereira Rossell (Montevideo), Dra MC Lapides, Hospital Paysandú (Paysandú).

## ISAAC Phase Three National Coordinators not identified above: Brazil

Professor D Solé, Universidade Federal de São Paulo; **Canada:** Professor MR Sears, McMaster University; Chile: Dra V Aguirre, Hospital CRS El Pino; **Fiji:** Dr L Waqatakirewa, Children’s Hospital; **India:** Dr J Shah, Jaslok Hospital & Research Centre; **Indonesia:** Prof Dr K Baratawidjaja, University of Indonesia; **Japan:** Professor S Nishima, The National Minami-Fukuoka Chest Hospital; **Mexico:** Dr M Baeza-Bacab, University Autónoma de Yucatán; **New Zealand:** Professor MI Asher, University of Auckland; **Singapore:** Professor B-W Lee, National University of Singapore.
